# Clinical-radiological predictive model in differential diagnosis of small (≤ 20 mm) solitary pulmonary nodules

**DOI:** 10.1186/s12890-021-01651-y

**Published:** 2021-09-05

**Authors:** Hai-Cheng Zhao, Qing-Song Xu, Yi-Bing Shi, Xi-Juan Ma

**Affiliations:** 1 Shuanggou Hospital Department, Xuzhou Central Hospital, 199 South Jiefang Road, Xuzhou, China; 2 Department of Radiology, Xuzhou Central Hospital, 199 South Jiefang Road, Xuzhou, China

**Keywords:** Small solitary pulmonary nodule, Diagnosis, Predictive model, Pre-test probability

## Abstract

**Background:**

There is a lack of clinical-radiological predictive models for the small (≤ 20 mm) solitary pulmonary nodules (SPNs). We aim to establish a clinical-radiological predictive model for differentiating malignant and benign small SPNs.

**Materials and methods:**

Between January 2013 and December 2018, a retrospective cohort of 250 patients with small SPNs was used to construct the predictive model. A second retrospective cohort of 101 patients treated between January 2019 and December 2020 was used to independently test the model. The model was also compared to two other models that had previously been identified.

**Results:**

In the training group, 250 patients with small SPNs including 156 (62.4%) malignant SPNs and 94 (37.6%) benign SPNs patients were included. Multivariate logistic regression analysis indicated that older age, pleural retraction sign, CT bronchus sign, and higher CEA level were the risk factors of malignant small SPNs. The predictive model was established as: X = − 10.111 + [0.129 × age (y)] + [1.214 × pleural retraction sign (present = 1; no present = 0)] + [0.985 × CT bronchus sign (present = 1; no present = 0)] + [0.21 × CEA level (ug/L)]. Our model had a significantly higher region under the receiver operating characteristic (ROC) curve (0.870; 50% CI: 0.828–0.913) than the other two models.

**Conclusions:**

We established and validated a predictive model for estimating the pre-test probability of malignant small SPNs, that can help physicians to choose and interpret the outcomes of subsequent diagnostic tests.

## Background

At present, chest computed tomography (CT) has been widely used for routine physical examination. Therefore, the solitary pulmonary nodules (SPNs) are often detected occasionally [1–10]. When the SPN is larger than 6 mm, regular CT follow-up is needed [10]. The probability of malignancy increases as the diameter of SPN increases [10]. Although video-assisted thoracic surgery (VATS)-guided wedge resection or CT-guided needle biopsy have been extensively used for SPNs’ diagnosis because of their high diagnostic accuracy [11–14]. Although these diagnostic approaches are mini-invasive, a comprehensive preoperative analysis of the SPNs is still needed.

Preoperative evaluation of SPNs has traditionally relied on physicians' and radiologists' knowledge. There is also a lack of reproducibility since one's knowledge of decision is invariably one-sided and closely linked to the doctor's realistic experience. To address this problem, researchers used a clinical-radiological predictive model to investigate the clinical characteristics of SPNs [15–17].

SPNs with a size ≤ of 20 mm are called small SPNs [18]. Although the size of the SPNs is an independent risk factor of malignancy [15–17], approximately 67.5–78% of small SPNs were malignant [18]. At present, many clinical-radiological predictive models for SPNs have been established around the world [15–17]. However, these models are not stratified by the size of SPNs. Predictive models for small SPNs are still lacking.

This study aimed to develop a clinical-radiological predictive model for distinguishing between malignant and benign small SPNs.

## Methods

This single-center study was approved by Ethics Committee of Xuzhou Central Hospital. Because of its retrospective nature, the need for written informed consent was waived by the Ethics Committee of Xuzhou Central Hospital. All methods were carried out in accordance with Declaration of Helsinki.

### Study design

From 2013 (January) to 2018 (December), consecutive patients with small SPNs were enrolled in this study as a training group for establishing a clinical-radiological predictive model. In the second phase of the study, consecutive patients with small SPNs from January 2019 to December 2020, were included as a validation group for examining the reliability of the model.

The inclusion was based on: (a) patients with SPNs; (b) diameter of SPN ≤ 20 mm; (c) solid SPNs; and (d) SPNs with a definite pathological diagnosis. Patients with any of these factors were excluded from the study i.e., (a) patients with malignant history within 5 years; (b) diameter of SPN < 5 mm; and (c) patients with incomplete clinical data.

The imaging features of SPNs were assessed on chest CT at the lung window (window level =  − 500 Hounsfield units [HU]; window width = 1200 HU) and at the mediastinal window (window level = 50 HU; window width = 450 HU). The CT parameters were kept as 120 kVp, 100–200 mAs, 0.75–1.5 pitch, and 0.625–1.25 mm collimation. With a thickness of 1.0––1.25 mm, the images were reconstructed employing a medium sharp (B50) reconstruction algorithm. The CT images were analyzed by two chest radiologists who were blinded to the pathological findings. The CT features of the SPNs included lobulation, spiculation, pleural retraction sign, CT bronchus sign, and calcification.

The patients' clinical data included their age, gender, malignant history, smoking history, and tumor marker levels.

### Pathological diagnosis

The pathological diagnosis of the malignant SPNs could be obtained via two ways i.e., (a) surgical resection; and (b) lung biopsy.

Similarly, the pathological diagnosis of benign SPNs could also be made via two ways i.e., (a) surgical resection; and (b) if the lung biopsy results indicated the specific benign results (benign tumors, mycotic infection, or tuberculosis), they could be accepted as the final diagnosis [18].

### Definitions [19, 20]

Lobulation is defined as a wavy or scalloped portion of the lesion's surface, and strands extending into the lung parenchyma from the nodule margin. The existence of strands spreading from the nodule margin into the lung parenchyma but not touching the pleural surface is referred to as spiculation. A linear attenuation going toward the pleura or the major or minor fissure from an SPN is known as a pleural retraction sign. The direct involvement of bronchiole in the nodules is known as the CT bronchus sign. Calcification is considered if the lesion has one of these patterns i.e., lamination, central nidus, diffusion, or popcorn pattern.

### Statistical analysis

For statistical analysis, SPSS 16.0 (SPSS Inc., Chicago, IL, USA) software was used. The data from the training group was analyzed using single-factor analysis to assess all factors that influence the probability of SPN malignancy. Then, to find independent prediction factors, multivariate logistic regression was used. The findings of the multivariate logistic regression were then used to construct a predictive model for SPN. The area under the curve (AUC) was determined and receiver operator characteristic (ROC) curves were created. A suitable cut-off point was calculated. The AUC of several ROC curves was compared using MedCalc statistical software (Ostend, Belgium). Statistical significance was described as a P value of less than 0.05.

## Results

### Training group

In the training group, 250 patients with small SPNs were included (Table 1). There were 156 (62.4%) malignant SPNs and 94 (37.6%) benign SPNs. The details of the pathological diagnoses of SPNs were shown in Table 2.

**Table 1:** Baseline data of the training group

	Malignant (n = 156)	Benign (n = 94)	P value
Gender (male/female)	85/71	56/38	0.432
Age (y)	65.4	53.5	< 0.001
Smoking history	72	38	0.377
Malignant history	5	1	0.519
*CT features*			
Diameter (mm)	16.8	13.6	< 0.001
Lobulation	114	49	0.001
Spiculation	120	47	< 0.001
Pleural retraction sign	75	17	< 0.001
CT bronchus sign	104	34	< 0.001
Calcification	3	9	0.006
Mediastinal/hilar lymph nodule ≥ 10 mm	38	14	0.074
*Lobes*			0.951
Upper	67	40	
Non-upper	89	54	
*Sides*			0.897
Left	76	45	
Right	80	49	
*Tumor markers*			
CEA (ug/L)	3.9	2.3	< 0.001
Cyfra21-1 (ng/ml)	13.5	12.7	0.099
SCC (ug/L)	1.4	1.1	0.211
NSE (ng/ml)	2.5	2.4	0.423

CEA: Carcinoembryonic antigen; CT: Computed tomography; NSE: Neuron-specific enolase; SCC: Squamous cell carcinoma antigen

**Table 2:** Pathological diagnoses in the training group

Malignant	156
Adenocarcinoma	112
Squamous cell carcinoma	33
Adenosquamous carcinoma	7
Small-cell lung cancer	4
Benign	94
Inflammatory pseudotumor	70
Hamartoma	11
Tuberculoma	8
Lymph nodule	4
Mycotic infection	1

### Predictive model

At univariate logistic analysis, older age, larger diameter, lobulation, spiculation, pleural retraction sign, CT bronchus sign, mediastinal/hilar lymph nodule ≥ 10 mm, and higher carcinoembryonic antigen (CEA) levels were the risk factors of malignant small SPNs. Calcification was a predictive factor for benign small SPNs (Table 3). When these factors were combined into the multivariate logistic analysis, we identified that older age (HR 1.138; CI (95%): 1.092–1.186; *P* < 0.001), pleural retraction sign (HR: 3.366; CI (95%) 1.431–7.920; *P* = 0.005), CT bronchus sign (HR: 2.608; CI (95%) 1.190–5.714; *P* = 0.017), and higher CEA level (HR 1.234; CI (95%) 1.032–1.475; *P* = 0.021) were the risk factors of malignant small SPNs (Table 3).

**Table 3:** Predictors of malignancy

Variables	Univariate analysis			Multivariate analysis		
	Hazard ratio	95% CI	*P* value	Hazard ratio	95% CI	*P* value
Age	1.120	1.085–1.157	< 0.001	1.138	1.092–1.186	** < 0.001**
Size	1.177	1.107–1.252	< 0.001	1.086	0.995–1.181	0.065
Lobulation	2.493	1.456–4.267	0.001	2.143	0.980–4.689	0.056
Spiculation	3.333	1.924–5.776	< 0.001	0.739	0.302–1.806	0.507
Pleural retraction sign	4.194	2.274–7.734	< 0.001	3.366	1.431–7.920	**0.005**
CT bronchus sign	3.529	2.064–6.035	< 0.001	2.608	1.190–5.714	**0.017**
Calcification	0.185	0.049–0.702	0.013	0.190	0.024–1.517	0.117
Mediastinal/hilar lymph nodule ≥ 10 mm	1.840	0.937–3.615	0.077	0.732	0.312–1.720	0.475
CEA	1.313	1.117–1.544	0.001	1.234	1.032–1.475	**0.021**

CEA: Carcinoembryonic antigen; CI: confident interval; CT: computed tomography

The clinical-radiological predictive model was made based on the risk factors mentioned above. Probability of malignant small SPNs: P = e^x^/(1 + e^x^).

X = − 10.111 + [0.129 × age (y)] + [1.214 × pleural retraction sign (present = 1; no present = 0)] + [0.985 × CT bronchus sign (present = 1; no present = 0)] + [0.21 × CEA level (ug/L)].

**Fig. 1:**
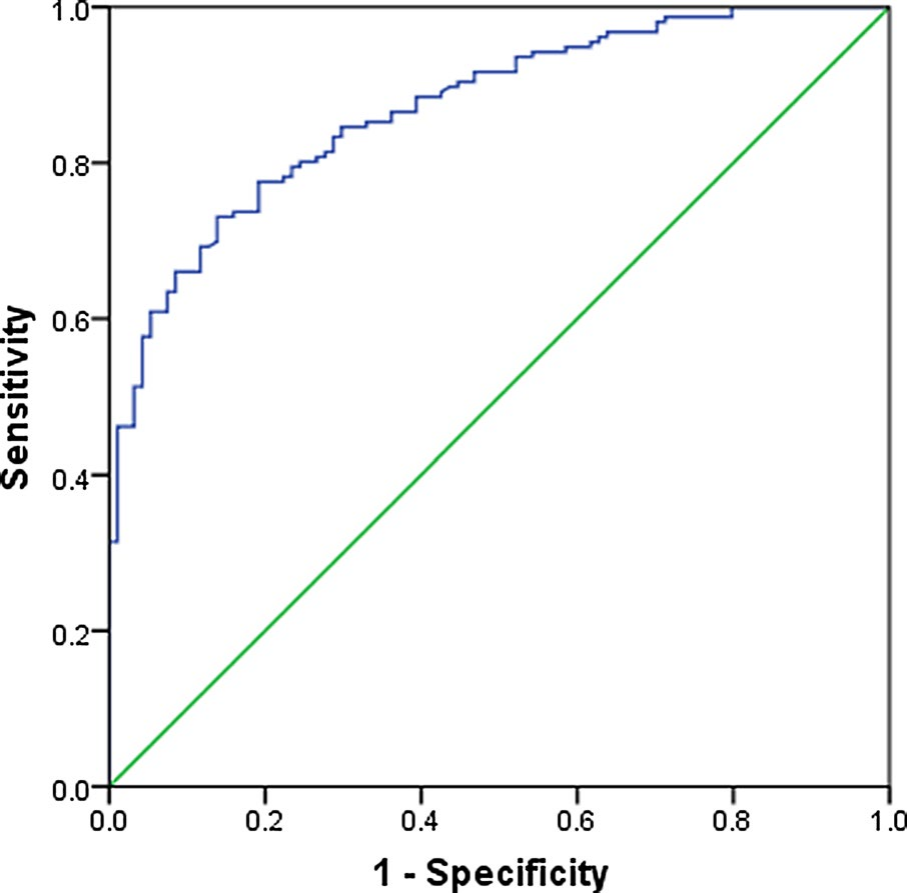
The ROC curve generated using the predictor from training group

The AUC of the ROC curve of this model was 0.870 (CI (95%): 0.828–0.913, P < 0.001, Fig. 1). To maximize sensitivity and specificity, we selected a cut-off risk score of − 1.1635 (sensitivity = 83.3%, specificity = 71.3%). If the score was greater than or equal to − 1.1635, the small SPN was considered to be malignant. If the score was less than − 0.1.759, the small SPN was considered to be benign.

### Validation group

In the validation group, a total of 101 patients with small SPNs (malignant: 64; benign: 37) were included. There was no significant difference between training and validation groups’ patient data (Table 4).

**Table 4:** Baseline data between the training and validation group

	Training (n = 250)	Validation (n = 101)	P value
Gender (male/female)	141/109	49/52	0.180
Age (y)	60.9	61.4	0.723
Smoking history	110	43	0.807
Malignant history	6	2	1.000
*CT features*			
Diameter (mm)	15.6	15.9	0.534
Lobulation	163	62	0.500
Spiculation	157	67	0.532
Pleural retraction sign	92	36	0.839
CT bronchus sign	138	58	0.704
Calcification	12	2	0.264
Mediastinal/hilar lymph nodule ≥ 10 mm	52	24	0.542
*Lobes*			0.705
Upper	107	41	
Non-upper	143	60	
*Sides*			0.390
Left	121	54	
Right	129	47	
*Tumor markers*			
CEA (ug/L)	3.3	3.2	0.886
Cyfra21-1 (ng/ml)	2.5	2.4	0.604
SCC (ug/L)	1.3	1.4	0.614
NSE (ng/ml)	13.2	13.1	0.890
*Final diagnoses*			0.865
Malignant	156	64	
Benign	94	37	

CEA: Carcinoembryonic antigen; CT: Computed tomography; NSE: Neuron-specific enolase; SCC: Squamous cell carcinoma antigen

### Test for the model

The validation group’s patient data was used to assess the accuracy of this model. To compare our model to other predictive models, we selected two other models made before. One was made in China by Wang et al. [21], and another was made in Western by Swensen et al. [22].

Wang et al. [21] model: X = − 4.8029 − [0.743 × gender (male = 1; female = 0)] + [0.057 × age (y)] + [1.306 × malignant history (present = 1; no present = 0)] + [1.305 × ground glass nodule (present = 1; no present = 0)] + [0.051 × diameter (mm)] + [1.043 × spiculation (present = 1; no present = 0)].

Swensen et al. [22] model: X = − 6.8272 + [0.0391 × age (y)] + [0.7917 × cigarettes history (yes = 1; no = 0)] + [1.3388 × malignant history (present = 1; no present = 0)] + [0.1274 × diameter (mm)] + [1.0407 × spiculation (present = 1; no present = 0)] + [0.7838 × upper (upper = 1; non-upper = 0)].

**Fig. 2:**
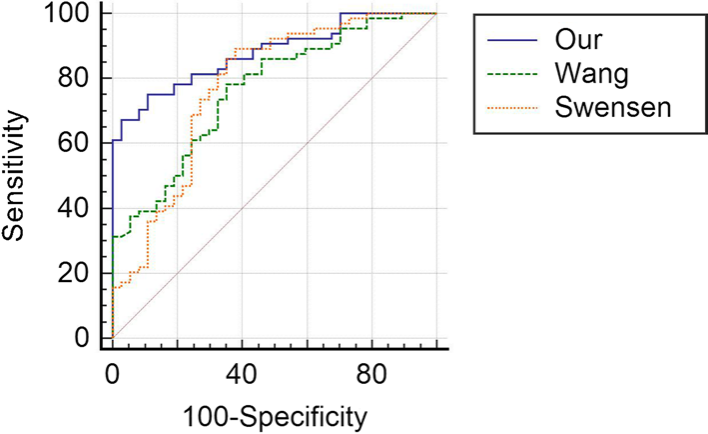
AUC comparison between our, Wang et al. and Swensen et al. models. The three ROC curves were generated using the data in validation group

When we put the data of patients in the validation group into Wang et al. [21], and Swensen et al. [22] models, the AUC of the ROC curves were 0.878 (CI (95%): 0.797–0.934), 0.763 (CI (95%): 0.668–0.842), and 0.775 (CI (95%): 0.681–0.852), respectively. The AUC under our model was significantly larger than that under Wang (P = 0.0029) and Swensen (P = 0.0315) models (Fig. 2).

## Discussion

This study found two clinical characteristics (i.e., age and CEA level) and two CT characteristics (i.e., pleural retraction sign and CT bronchus sign) as significant predictors of malignancy in patients with small SPNs. Notably, we developed a clinical-radiologic predictive model to estimate the pre-test patient-specific “risk” of malignant small SPNs with good predictive accuracy.

In most of the previous models, they usually included 6–7 predictive factors [15, 20–22]. However, our model only included 4 predictive factors. Compared to the previous models, the diameter of the nodule, spiculation, and malignant history were not associated with the malignant small SPNs.

The SPNs’ malignancy risk increased 1.1 times as the nodule diameter increased by 1 mm, according to She et al. [20]. However, the diameter of SPN might have a small impact on the differential diagnosis of small SPNs. Chen et al. [23] also did not find the malignancy of small SPNs was associated with the diameter. Since the risk of malignancy increases with larger SPNs, models for 20–30 mm SPNs can outperform those for 20 mm or fewer counterparts in terms of predicting it [23].

Spiculation is a common CT sign which indicates malignant SPN [15, 20–23]. However, previous studies did not analyze the detailed features of the spiculation. Some researchers found that both lung cancer and inflammatory pseudotumor could be presented with spiculation [24]. However, the morphology of the spiculation in lung cancer and inflammatory pseudotumor were different [24]. The lung cancers are usually presented with short spiculation, while the inflammatory pseudotumors are mostly presented with long spiculation [24]. However, the morphology of the spiculation was usually confirmed according to the doctor’s practical experience and there were no strict definitions of short and long spiculation. As a result, the presence or absence of spiculation was usually a binary parameter (present/absent), with no set threshold for distinguishing between these criteria. Previous malignant history is also an important factor of malignant SPNs, however, our study only had 6 patients with a malignant history. Therefore, malignant history was not found to be associated with malignant SPNs in our study.

Similar to other models [20–23], our model also included age, pleural retraction sign, CT bronchus sign, and CEA level as the predictors of malignant SPNs. Lung cancer onset before 30 years of age is extremely rare, according to Chen et al.'s [25] study in China; however, its incidence increases gradually between the ages of 30–75.

Pleural retraction sign also could be found in many predictive models [17, 19, 20, 23]. According to a previous report, 18 of the 29 cases of malignant nodules had pleural retraction sign, and the rest of the cases without pleural retraction sign had malignant nodules distant from the pleura [26]. According to Li et al. [26], the frequency of pleural retraction sign is 13.1% in benign nodules and 25.4% in malignant nodules. Furthermore, Cui et al. [27] found that diagnosing lung cancer with only pleural retraction sign is not specific, while pleural retraction sign paired with an associated notch has a specificity of 96% in diagnosing lung cancer, with a positive prediction rate of 97%.

The presence of air bronchus within SPN lesions is referred to as the CT bronchus sign. According to Ma et al. [28], the incidence of CT bronchus sign for adenocarcinoma is as high as 48.8%, while undifferentiated carcinoma, squamous carcinoma, and alveolar carcinoma are 28.6%, 20%, and 9.1% respectively.

Furthermore, serum tumor markers have been linked to cancer [29], and CEA has been an essential marker for various cancers [30]. Even though serum CEA levels were linked to age and smoking [30], multivariate analysis revealed serum CEA to be a significant factor instead of a confounding factor. CEA was also thought to be a key factor in distinguishing between malignant and benign SPNs by Li et al. [30].

When comparing our model to Wang et al. [21] and Swensen et al. [22] models, we found that the AUC was significantly larger in our model. Both Wang et al. [21] and Swensen et al. [22] included SPNs with a diameter ≤ of 30 mm. These results indicated that most previous models might not be suited for the small SPNs. However, we only focused on the SPNs with a diameter ≤ of 20 mm (small SPNs). Therefore, our model improved the diagnostic ability for the small SPNs.

This study has some limitations. Since this was a single-center retrospective study, the accuracy and reliability of our prediction model need to be validated in a multi-center prospective study before it can be used as a clinical tool for the prediction of small SPNs’ malignancy. Besides, many studies also utilized CT follow-up as a reference standard for benign SPNs [18, 22, 31]. However, we only included the SPNs with the pathological diagnoses. This performance decreased the number of benign SPNs and might influence the results of risk factors. However, the pathological results could guarantee the accuracy of the diagnoses of SPNs. Moreover, FDG-PET scans are often useful in the diagnosis of lung cancer, and they are now widely practiced in some developed countries. However, since FDG-PET is not accessible to all patients in China and we do not have complete data, thus our model's generalizability may be limited.

## Conclusion

In conclusion, older age, pleural retraction sign, CT bronchus sign, and higher CEA levels were independent predictors of malignancy in patients with small SPN. Moreover, we developed a predictive model that approximates patient-specific “risk” of malignant small SPN with good predictive accuracy, thus can assist in selecting and interpreting the results of subsequent diagnostic tests.

## Data Availability

The data that support the findings of this study are available from the corresponding author upon reasonable request.

## Abbreviations

AUC: Area under the curve
CT: Computed tomography
CEA: Carcinoembryonic antigen
ROC: Receiver operator characteristic
SPN: Solitary pulmonary nodule
VATS: Video-assisted thoracic surgery
